# Detection of respiratory syncytial virus based on RT-RPA and CRISPR-Cas12a

**DOI:** 10.3389/ebm.2025.10387

**Published:** 2025-05-01

**Authors:** Ariya Khamwut, Juthamas Nimnual, Nantinee Chomta, Pattaraporn Nimsamer, Oraphan Mayuramart, Pornchai Kaewsapsak, Siripat Pasittungkul, Yong Poovorawan, Sunchai Payungporn

**Affiliations:** ^1^ Center of Excellence in Systems Microbiology, Faculty of Medicine, Chulalongkorn University, Bangkok, Thailand; ^2^ Division of Medical Bioinformatics, Research Department, Faculty of Medicine Siriraj Hospital, Mahidol University, Bangkok, Thailand; ^3^ Department of Biochemistry, Faculty of Medicine, Chulalongkorn University, Bangkok, Thailand; ^4^ Center of Excellence in Clinical Virology, Faculty of Medicine, Chulalongkorn University, Bangkok, Thailand

**Keywords:** CRISPR-Cas12a, detection, isothermal amplification, RT-RPA, respiratory syncytial virus

## Abstract

Human respiratory syncytial virus (hRSV) is one of the most prevalent viruses infecting children globally. In this study, we employed the RT-RPA with CRISPR/Cas12a detection methodology to detect and differentiate RSV-A and RSV-B, particularly in resource-limited settings. The detection limit for RSV-A and RSV-B was approximately 10^2^ and 10^3^ copies/reaction, respectively. The assay revealed 100% specificity in detecting both RSV-A and RSV-B. Diagnostic accuracy was 90.32 and 93.55% for RSV-A and RSV-B, respectively, compared to RT-qPCR. These data indicate a proficient strategy for RSV screening, demonstrating promise for prospective applications in detecting diverse viral infections.

## Impact statement

This study presents a novel RSV detection method using RT-RPA and CRISPR/Cas12a, achieving high sensitivity and specificity. With detection limits of 10^2^ and 10^3^ copies/reaction for RSV-A and RSV-B, respectively, and diagnostic accuracy comparable to RT-qPCR, it offers a reliable, resource-efficient alternative for accurate RSV screening.

## Introduction

The human respiratory syncytial virus (hRSV) is an enveloped (−) ssRNA virus belonging to the *Paramyxoviridae* family and is classified in the genus *Pneumovirus*. RSV has a single serotype divided into two major subgroups, RSV-A and RSV-B, each comprising multiple genotypes [1–3]. The replication of hRSV occurs in both the upper and lower airways and is commonly disseminated through direct contact or aerosol inhalation [2, 4]. The predominant clinical presentation of RSV infection observed in children is bronchiolitis, a pathological condition affecting the lower respiratory tract, marked by airway obstruction. This condition may progress to complications such as pneumonia, respiratory failure, and mortality [5, 6].

Presently, preventive strategies rely on avoiding risk factors, patient isolation, and vaccination. However, existing vaccines primarily offer inadequate protection against RSV infection in older adults and high-risk individuals. Consequently, rapid diagnosis of RSV infection is essential to track and reduce the spread of diseases. The World Health Organization (WHO) advocates the utilization of a virus testing approach employing quantitative reverse transcription-polymerase chain reaction (qRT-PCR) conducted on throat or nasopharyngeal swabs (NP) [7, 8]. However, the use of qRT-PCR remains restricted because of the high cost of the devices, the need for a specialized molecular lab and specialists, and the 4-h detection turnaround time.

Interestingly, the U.S. Food and Drug Administration (FDA) recently approved the CRISPR-based diagnostic approach for more straightforward and rapid diagnosis [9]. This approach integrates the CRISPR/Cas system with recombinase polymerase amplification (RPA) to amplify and identify target regions within DNA sequences [10, 11]. The CRISPR-Cas12a technology, with its portable, rapid, and precise characteristics, holds immense promise in nucleic acid diagnostics, potentially transforming the field of virology and molecular diagnostics [12].

CRISPR-Cas12a, a CRISPR nuclease, possesses the capability to selectively degrade designated double-stranded DNA (dsDNA) and single-stranded DNA (ssDNA) that is complementary to the CRISPR RNA (crRNA). This process involves the utilization of the RuvC domain, resulting in the creation of staggered ends in the target DNA (*cis*-cleavage activity) after recognition of the protospacer adjacent motif (PAM) (5′-TTTV-3′) [13, 14]. After identifying the corresponding sequences to the CRISPR RNA (crRNA), the Cas protein cleaves the specific target. A matching DNA sequence triggers the collateral effect (*trans*-cleavage activity) of Cas12a. This activation separates the fluorescent reporter from the quencher, illuminating the fluorescent signal [15].

Despite extensive research on the CRISPR-Cas12a technology for the expeditious and precise detection of human respiratory viruses, there remains a constrained capacity for nucleic acid identification of RSV in clinical samples in relation to both subtypes, A and B. This limitation underscores the need for a more advanced and specific diagnostic tool. Therefore, the objective of this study is to establish an RT-RPA with a CRISPR/Cas12a-based technique specifically designed to identify RSV-A and RSV-B, a breakthrough that could revolutionize RSV diagnostics.

## Materials and methods

### Design and selection of primers and crRNA for specific RSV detection

Primers specific to the glycoprotein (G) genes of the human respiratory syncytial virus (RSV) and CRISPR RNA (crRNA) were meticulously designed to target the conserved region, and the sequences were selected from the NCBI database^1^. In designing crRNA, the selected sequences were adjacent to the PAM sequence (TTTV) [13], and the scaffold and T7 promoter sequence were then added to the 5′-end. This careful design process ensures the specificity and accuracy of our diagnostic tool. All primers and crRNAs used are presented in Table 1.

**TABLE 1:** Oligonucleotides used in the study.

Assay	Primer name	Sequence (5´ ⟶ 3′)	Length (bp)	Product size (bp)
RT-RPA	RSVA-G-F282	ATA​CCT​CAC​CCA​GAA​TCC​CCA​GCT​TGG​AAT	30	391
	RSVA-G-R672	GGTTTGAGGTTTGRGATCTTTTTTGGTTGTCTT	33	
	RSVB-G-F39	CAC​TGC​CAG​TAC​TCT​AGA​AAA​GAC​CTG​GGA	30	384
	RSVB-G-R422	CTG​CCT​TTG​GTT​TGT​GCT​GTT​GTA​TGG​TGT	30	
crRNA	RSVA-G598	UAA​UUU​CUA​CUA​AGU​GUA​GAU​CAG​GUU​UUU​UGU​UUG​GUA​UU	41	
	RSVB-G154	UAA​UUU​CUA​CUA​AGU​GUA​GAU​GCA​AUG​AUA​AUC​UCA​ACC​UC	41	

Note: The underline represents the spacer sequence.

### crRNA construction and purification

For crRNA construction, the annealing reaction consisted of 10 μL of a 2.5 μL of 10x ligation buffer, 10 μM of DNA template for crRNA, 10 μL of a 10 μM T7 promoter primer, and 2.5 μL of DEPC. The annealing process was as follows: 95°C for 3 min, 65°C for 3 min, 42°C for 5 min, and 37°C for 45 min. Subsequently, crRNA transcription was executed utilizing the RiboMAX™ Large Scale RNA Production System (Promega, United States), following the manufacturer’s prescribed protocol. Post-transcription, the crRNA, consisting of approximately 40 nucleotides, underwent purification employing a miRNA isolation kit (Geneaid, Taiwan) and was subsequently quantified using the Qubit™ microRNA Assay Kit (Thermo Fisher Scientific, United States).

### RT-RPA reaction setup and protocol

A commercial reverse transcription recombinase polymerase amplification (RT-RPA) kit (TwistDx, UK) was employed to perform the isothermal amplification of the RSV-A and RSV-B genes. In brief, the RT-RPA reaction was constituted by combining 29.5 μL of rehydration buffer, 2.4 μL of a 10 μM forward primer, 2.4 μL of a 10 μM reverse primer, 1 μL of 200 U/μL Reverse Aid RT (Thermo Fisher Scientific, United States), 4.2 μL of DEPC, and 1 μL of the RNA template. Subsequently, 280 nM of MgOAC was introduced to the cap, and the mixture was vortexed and spun down for 2 min. The mixture was incubated in a mini heating dry bath (Major Science, United States) at 39°C for 30 min, followed by a subsequent heat inactivation step at 75°C for 5 min.

### CRISPR/Cas12a cleavage assay procedure

Next, the CRISPR/Cas12a cleavage assay was conducted within a 15 μL reaction system. The mixture comprised 1.5 μL of 10x NEB buffer 2, 1.5 μL of 300 nM crRNA, 0.5 μL of 1 μM Cas12a (New England Biolabs, United States), 0.5 μL of 6 μM ssDNA probe labeled with FAM and BHQ1, 10 μL of DEPC, and 1 μL of RT-RPA template. Subsequently, the reaction mixtures underwent incubation in a mini heating dry bath (Major Science, United States) at 39°C for 15 min. The fluorescent signal was observed on a BluPAD Dual LED Blue/White Light Transilluminator (Bio-Helix, Taiwan) by the naked eye. The assay was conducted in triplicate, and the findings were based on consensus interpretations derived from three interpreters. The criteria for interpretation by the naked eye include the absence of fluorescence in negative samples, and a clear fluorescence or color change in positive samples. Positive results should be visibly distinguishable from negative controls, with the test sample showing a stronger signal than the background or controls.

### Determining the limit of detection for RT-RPA and CRISPR-Cas12a

To determine the limit of detection, standard RNA was generated using RiboMAX™ Large Scale RNA Production System (Promega, United States) according to the manufacturer’s protocol. Then, DNase I (Thermo Fisher Scientific, United States) was used to eliminate the DNA templates. Next, the synthesized standard RNA was purified and quantified using a miRNA isolation kit (Geneaid, Taiwan) and Qubit™ microRNA Assay Kit (Thermo Fisher Scientific, United States), respectively. Using a 10-fold serial dilution, synthetic standard RNAs with copy numbers ranging from 10^7^ to 10 copies/μL were prepared. The limit of detection was determined by the RT-RPA followed by CRISPR-Cas12a detection. The fluorescent signal was observed on a BluPAD Dual LED Blue/White Light Transilluminator (Bio-Helix, Taiwan) by the naked eye.

### Cross-reactivity testing with various respiratory viruses

Next, cross-reactivity testing was conducted on diverse human respiratory viral samples, including influenza viruses A (A/H1 and A/H3), influenza viruses B (B/Yam and B/Vic), human bocavirus (HBoV), coronavirus (CoV), human metapneumovirus (HMPV), human parainfluenza virus (HPIV), and human rhinovirus (HRV). This assessment was carried out utilizing RT-RPA and CRISPR-Cas12a detection methodologies (n = 10 for each).

### Calculation of sensitivity, specificity, and accuracy

The sensitivity, specificity, and accuracy were calculated using the web-based diagnostic test evaluation calculator MedCalc software^2^ [16]. This web-based tool was utilized to calculate these metrics, ensuring a comprehensive analysis of the test performance. The formulas for calculating sensitivity, specificity, and accuracy are as follows:

$$\text{Sensitivity}=\frac{\text{True}\;\text{Positives}\;\left(\text{TP}\right)}{\text{True}\;\text{Positives}\;\left(\text{TP}\right)+\text{False}\;\text{Negatives}\left(\text{FN}\right)}$$

$$\text{Specificity}=\frac{\text{True}\;\text{Negatives}\;\left(\text{TN}\right)}{\text{True}\;\text{Negatives}\;\left(\text{TN}\right)+\text{False}\;\text{Positives}\;\left(\text{FP}\right)}$$

$$\text{Accuracy}=\frac{\text{True}\;\text{Positives}\;\left(\text{TP}\right)+\text{True}\;\text{Negatives}\left(\text{TN}\right)}{\text{True}\;\text{Positives}\;\left(\text{TP}\right)+\text{True}\;\text{Negatives}\left(\text{TN}\right)+\;\text{False}\;\text{Positives}\;\left(\text{FP}\right)+\text{False}\;\text{Negatives}\left(\text{FN}\right)\;}$$

### RNA sample collection and verification

The RNA samples were obtained from the Center of Excellence in Clinical Virology, Chulalongkorn University. Briefly, nasopharyngeal swab samples (n = 93) were collected from patients with influenza-like illness (ILI). The viral RNA was extracted using a GenUP™ Virus RNA kit (Biotech Rabbit, German). The RSV-positive sample was verified by RT-qPCR as described in the previous study [17] (cut-off at Ct ≤ 38).

## Results and discussion

**FIGURE 1:**
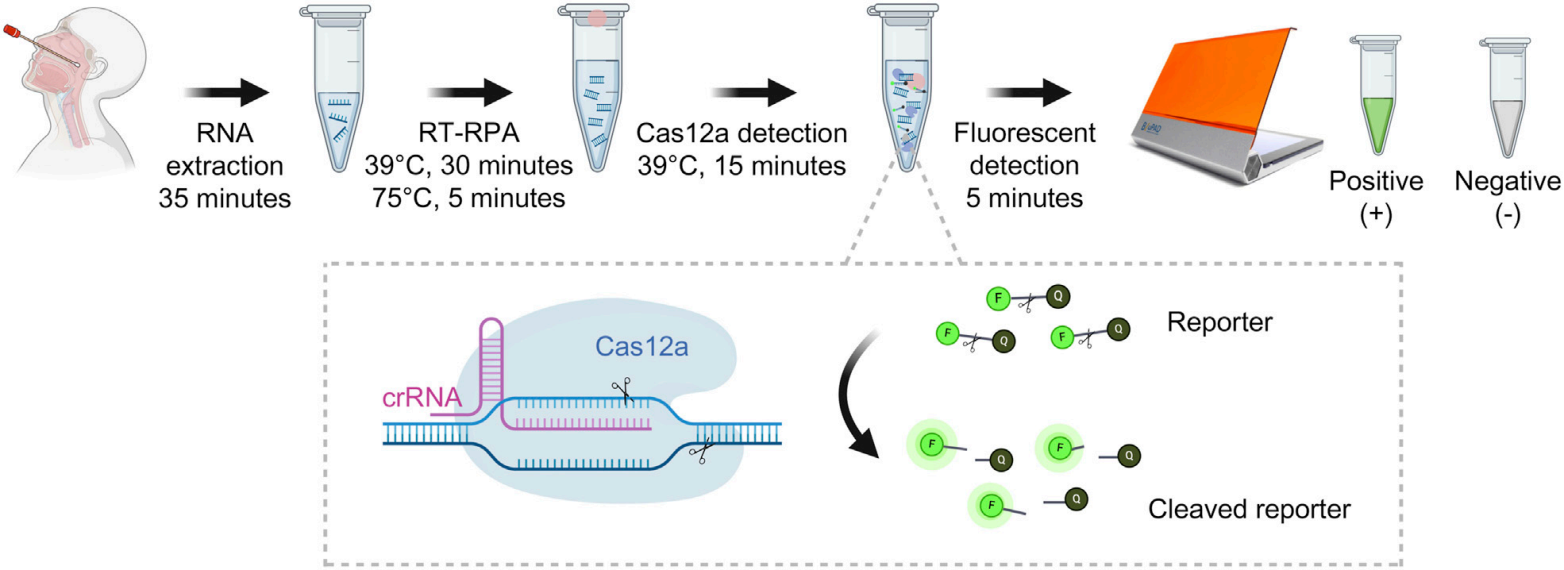
Detection of RSV-A and RSV-B based on RT-RPA and CRISPR-Cas12a (This figure was created with Biorender.com).

A recently developed approach for pathogen detection incorporating isothermal amplification techniques (such as RPA) coupled with fluorescent detection utilizing the CRISPR-Cas system [10, 18, 19] has been developed and shown to be useful for higher-sensitivity nucleic acid pathogen identification [11, 20]. Our experimental protocol procedure took approximately 90 min, covering the entire process from RNA extraction to fluorescent detection (Figure 1). Meanwhile, the qRT-PCR revealed an approximately 4-h turn-around time and required a specialized molecular laboratory, expensive equipment, and well-trained staff.

**FIGURE 2:**
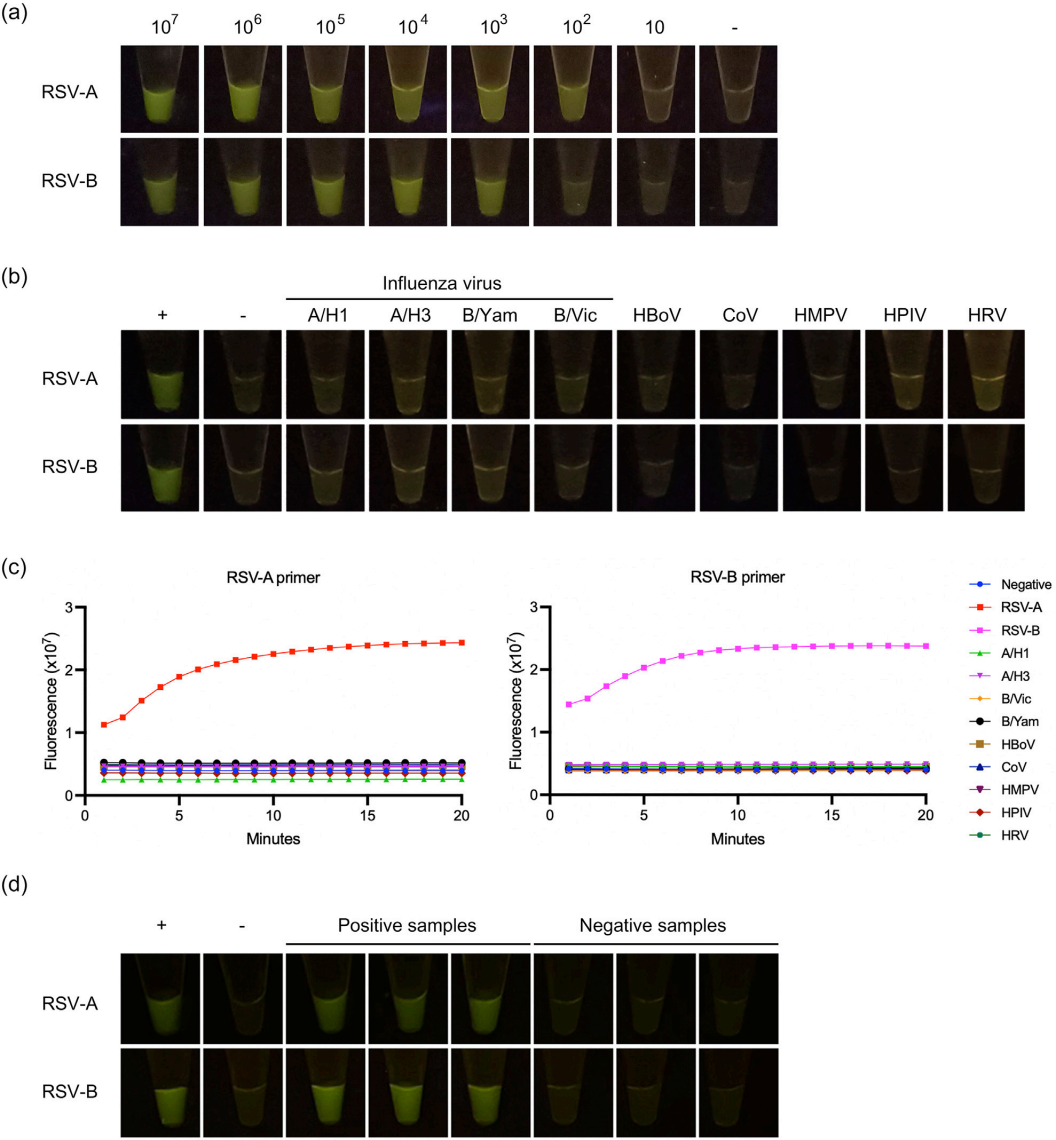
Detection of RSV-A and RSV-B based on RT-RPA and CRISPR-Cas12a. **(A)** The limit of detection. **(B)** The cross-reactivity testing against influenza viruses A (A/H1 and A/H3), influenza viruses B (B/Yam and B/Vic), human bocavirus (HBoV), coronavirus (CoV), human metapneumovirus (HMPV), human parainfluenza virus (HPIV), and human rhinovirus (HRV). **(C)** Cross-reactivity testing against other respiratory viruses was performed by qPCR using RT-RPA + CRISPR with RSV-A primers and RT-RPA + CRISPR with RSV-B primers. Dot plots show fluorescence intensity measurements taken from 1 to 15 min. **(D)** The representative of positive and negative detection.

A previous study has shown that RSV can be detected using the RPA coupled with the CRISPR-Cas approach at 10^4^ copies/mL [21]. As illustrated in the results (Figure 2A), the LOD for RSV-A and RSV-B were estimated to be around 10^2^ and 10^3^ copies per reaction, respectively. The outcomes demonstrated that the primers and crRNA used in this investigation exhibited notable specificity against RSV-A and RSV-B viruses (Figure 2B). No cross-reactivity was discerned with other respiratory viruses (Figure 2B). Interestingly, our study can discriminate between the RSV-A and RSV-B subtypes (Figure 2C). In addition, RT-RPA with CRISPR-Cas12a assay was performed using RSV-A primers on RSV-B targets, and *vice versa*. The results showed that the primers could not amplify across subtypes (Supplementary Figure S1). The representative detection results were shown in Figure 2D.

In this study, the RSV-positive sample was verified by RT-qPCR. The results showed 39 and 27 RSV-A and RSV-B positive samples, respectively. Prior investigations indicated that RSV-A exhibited specificity and sensitivity values of 73.08% and 90%, respectively, while RSV-B demonstrated specificity and sensitivity figures of 42.86% and 93.33%, respectively [22]. Our study suggests a modest enhancement in the specificity (100%) of RSV-A and RSV-B detection compared to the gold-standard detection outcomes from RT-qPCR, as shown in Table 2. However, the current study demonstrates lower sensitivity, with values of 76.92% and 77.78% for RSV-A RSV-B, respectively (Table 2). In the case of RSV-A, the findings revealed 30 true positives, 0 false positives, 9 false negatives, and 54 true negatives. Conversely, for RSV-B, the results indicated 21 true positives, 0 false positives, 6 false negatives, and 66 true negatives. The undetected positive samples were associated with high Ct (cycle threshold) values, which could indicate a lower viral load in these samples. Furthermore, since multiple freeze-thaw cycles and storage may have caused RNA degradation. Additionally, the diagnostic accuracy for RSV-A and RSV-B was reported as 90.32% and 93.55%, respectively.

**TABLE 2:** The performance of RT-RPA and CRISPR-Cas12a based assay.

Parameters	RSV-A	RSV-B
Total samples	93	93
True positive	30	21
True negative	54	66
False positive	0	0
False negative	9	6
Sensitivity	76.92%	77.78%
Specificity	100%	100%
Diagnostic accuracy	90.32%	93.55%

However, this research still has some limitations, including the challenges related to nonspecific amplification, variable sensitivity, isothermal conditions, guide RNA specificity, and the subjective nature of the readout. These highlight the importance of ongoing research and optimization for their practical and reliable application in diagnostics.

In conclusion, the benefits of this study include simplicity, quick turnaround time, affordability, and applicability in resource-constrained places. The assays would be helpful and appealing for systematic retesting of individuals and large-scale screening across many communities to isolate patients and prevent the spread of RSV.

## Data Availability

The original contributions presented in the study are included in the article/Supplementary Material, further inquiries can be directed to the corresponding author.

## Footnotes

1
https://www.ncbi.nlm.nih.gov/nuccore

2
https://www.medcalc.org/calc/diagnostic_test.php
