# Supplementary material for: Detection of respiratory syncytial virus based on RT-RPA and CRISPR-Cas12a
Source: Exp Biol Med (Maywood). 2025 May 1;250:10387. doi: 10.3389/ebm.2025.10387 (PMC12078183; doi:10.3389/ebm.2025.10387)
Supplement: Supplementary file 1 [file DataSheet1.pdf]

Supplementary materials

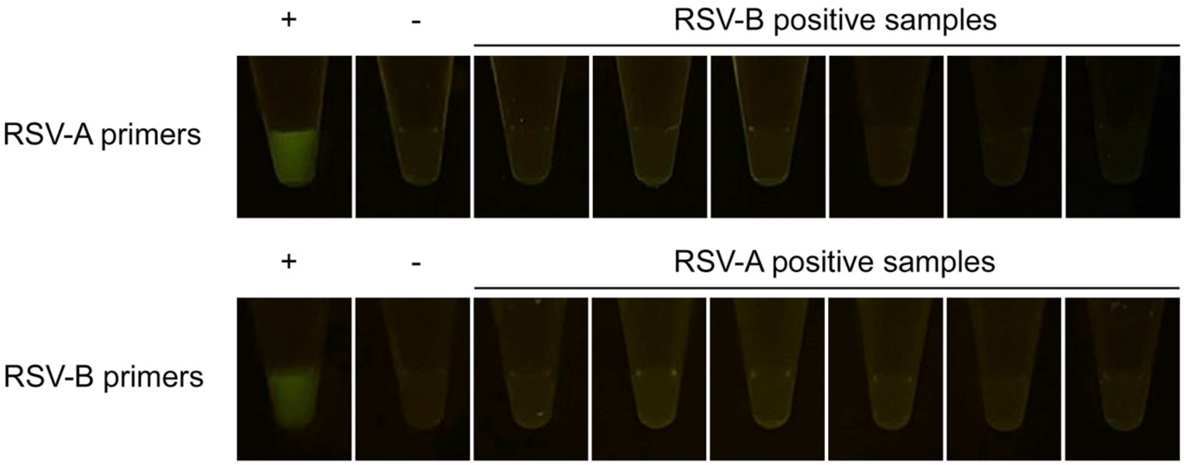

Supplementary Figure S1. The representative results of each set of primers when used against the other subtype. RT-RPA + CRISPR using RSV-A primers on RSV-B targets, and vice versa.
